# Fakes and chemicals: indigenous medicine in contemporary Kenya and implications for health equity

**DOI:** 10.1186/s12939-020-01313-1

**Published:** 2020-11-07

**Authors:** Olivia Howland

**Affiliations:** 1 PDRA, University of Liverpool, Liverpool, UK; 2 Visiting Scientist, ILRI, Nairobi, Kenya

**Keywords:** Kenya, Herbal medicine, Indigenous healing, Indigenous knowledge, Human medicine, Veterinary medicine, Counterfeit drugs, One Health, Trust, Chemicals

## Abstract

**Background:**

Access to effective biomedical treatments for humans and livestock in Kenya is far from universal. Indigenous healing has a significant role to play in contemporary society in Kenya, yet access is not the only reason for this. Beliefs surrounding the composition of biomedicines, people’s experiences of biomedical care, and issues of counterfeit biomedicines sold over the counter have led to patients seeking care outside of biomedical institutions.

**Methods:**

This study explores local realities of treatment seeking in one rural and one urban study site, for both humans and their livestock, including when, where and how people access certain types of medicines. Using an ethnographic approach to interviews, focus groups and observations, I explore the role that indigenous healing, both herbal and occasionally spiritual, plays within this context with healers and community members.

**Results:**

Indigenous healing remains important for many people due to their mistrust and suspicion of biomedicine and big pharma. Their interactions with the healer or doctor, and the equity of these interactions, influence their decisions whether to access herbal or biomedical care, or a combination of the two. Indigenous healing bridges the gap many people experience when they are unable to access biomedical treatments and effectively creates a broader, more equitable coverage for healthcare. The plurality of reasons surrounding decision making is complex, but it is clear that many people often use indigenous healing, improvements in the regulation of both formulas and practice would assist people to access more effective treatment.

**Conclusions:**

Indigenous healing is an important way in which Kenyans in rural and urban areas access healthcare for themselves and their animals. Issues of counterfeit biomedicines have led to broad mistrust and people favour indigenous healing, depending on the illness or severity of symptoms. Indigenous healing is a vital way in which people in underserved rural and urban populations access care. Herbal medicines and indigenous healing are trusted due to the greater transparency in their creation, and the more equitable relationship between indigenous doctor and patient. The study demonstrates that a pluralistic system is appropriate to increasing equity in access to healthcare in both urban and rural settings, as well as the importance of biomedical care providers respecting indigenous healing and viewing it with legitimacy. By taking a One Health perspective to understand the intersection of humans, livestock and the environment, we can better understand critical aspects affecting decision making for treatment and implications for healthcare equity in a rapidly changing world.

## Introduction

Indigenous,^1^ herbal and complimentary medicines are an important part of health care systems worldwide [1, 2]. In recent years there has been a global movement of a return to natural remedies and people have turned away from biomedical treatment, with rapid re-uptake of indigenous healing in many countries [1]. It is thought that 80 % of African populations use indigenous healing [3], helping to achieve universal coverage of access to healthcare and creating pluralistic systems of care where access to biomedical treatment is an issue [1, 4–7]. In addition to human use of herbs as medicine, it is also known that animals self-medicate with herbs [3]. Despite increasing equitable access to healthcare services, indigenous healing has been considered by certain groups of people including religious and socially conservative groups to be backward, ineffective, witchcraft, and dangerous [4]. Fewer studies explore the use of different medicines in conjunction with each other, but it is known that people use multiple sources of medicine at any one time [4, 8–13]. The global ‘herbal renaissance’ [5] is not absent from an African context [1] but understanding this intersection with biomedical care is less well understood [2].

Existing data suggest that use of healthcare services is purely an issue of access: the ratio of traditional healers to patients in Africa as a whole is 1:500, whereas for biomedical doctors this is 1:40,000 [4]. However, the rationale behind medical choices are complex and this complexity is underexplored in the literature [14, 15]. The choice of healers depends on the etiology of the condition based on local understandings of disease. For example, certain traditional healers could be visited when the cause of the condition is social rather than physiological. Medical decisions also vary across settings: rural inhabitants are known to utilise traditional healers more than urban inhabitants [16].

Lack of access to care heavily impacts equity: in Mathare, Nairobi, the ratio of traditional healers to population and of medical doctors is very similar (1:833 and 1:987 respectively), but in a rural location this ratio is dramatically different: 1 traditional doctor per 143 people, versus 1 biomedical doctor to 70,000 people [4]. The available data at a household level are limited, outdated [8], and what we do not understand well is what this means for health seeking behaviours.

The way in which people make the decision between using biomedicine or an indigenous one is complex [14]. There is a paucity of recent data available on this, especially so in African contexts. A study from Burkino Faso is one exception, finding that herbal medicine, self-administered, was the second most common form of healthcare in the study region. Visiting a hospital for allopathic care was the least common form of health seeking behaviour [8]. More than half of the study participants used indigenous healing at some point during their treatment regime of the most recent illness they had suffered [8].

Many people in Kenya access medicines over the counter from pharmacies, but available data on this is limited [10]. Some studies [12, 15] argue that the price of medicine is a key factor in people’s use of public (or state) health facilities, although sometimes there are other reasons for this. Counterfeit medicines are a significant issue in Kenya due to poor enforcement of the law and poor understanding of what counterfeit medicines are [11], indicating that trust may be an important factor in patient preference.

The qualitative study presented in this paper aims to provide a nuanced description of health decision making and the relationship between biomedical and indigenous healing to think about the challenges of achieving health equity in contemporary Kenya. This study would enable us to comprehend patient’s preferences and identify unmet health needs and the challenges of reaching health equity in healthcare settings. This is particularly relevant for LMICs where traditional medical practices coexist with biomedical ones [12]. In this paper I will present research findings from one urban and one rural Kenyan study site. The research explores healing for humans and livestock, and issues of access and use of medical care. It explores tensions between biomedical and indigenous healing in contemporary Kenya, issues of beliefs and practices, and the extant knowledge of plant-based medicines. It aims to understand the role that indigenous healing plays in providing equitable access to medicines and healing, patient preferences, and experiences of healthcare for people and their livestock.

## Background

Assumptions that biomedical care is the most reliable, legitimate and effective form of health care for both humans and livestock are prevalent in the existing literature and debates [13], yet there is a history of concerns around biomedical care which is based on both real and perceived risk [14]. Perhaps the most well-known example is controversy over vaccinations, and the rise of the antivax movement, which continues to gain momentum. Historical doubts and controversies have been perpetuated in the media, leading to hesitancy in uptake of important vaccines such as the MMR vaccine [15].

There have been incidents of vaccines going wrong and The Cutter Incident in 1955, where a polio vaccine contained live polio and killed several children, is one example [15]. In 1990 in Cameroon a rumour that routine childhood vaccines were being used to sterilize people led to a dramatic drop in uptake of vaccination programs [15]. There is also a history of unethical drug trials in LAMIC contexts, where patients are ill-informed and regulation is poor [17], causing further mistrust of biomedicine.

Indigenous healing has always been prevalent across the African continent, especially where there is inequitable healthcare coverage [12, 18]. In Kenya, people use biomedical and herbal medicines concurrently, but this has been reported as a barrier to effective treatment of serious illnesses [19]. Fear of safety and ideas of efficacy related to biomedicine [19], as well as indigenous healing bridging the gap in equity left by biomedicine, are among the reasons for use [13, 19]. The services of indigenous healers are not only sought by poor families: wealthy families in Burkina Faso who could afford biomedicine were regular users. Notably from a One Health perspective, the same study noted the importance of environmental resources for wellness, and specifically access to forests or areas rich in medicinal herbs [8].

Ethnoveterinary knowledge is vital for many livestock owners across Africa, due to difficulty in accessing veterinary medicines mostly as a result of the travelling expenses the livestock owner would need to incur to go from geographically remote areas to bigger towns [20]. Ethnoveterinary medicine is often effective, accessible and parallels Western techniques [21]. Many urban African settlements are home to livestock, and not only goats and chickens but cattle too, despite the challenge of space [22]. Ethnoveterinary medicine is important in these contexts too, particularly where costs of veterinary medicines are prohibitive, in order to provide broader coverage of care.

As a continent, Africa is rapidly urbanising and some believe that this urbanisation will lead to a loss of indigenous knowledge [22]. Studies which shed light on how urban populations use herbal medicines despite access to biomedicines are uncommon [7] and still suggest that those who use indigenous medicine have poor levels of education, indicating that it is still the preserve of the illiterate and ignorant. In South Africa, herbal and complimentary medicine has been effectively integrated into biomedical healthcare delivery systems and has contributed to the equitability of health and wellness of the nation, regardless of social or educational status [23].

Migration from one’s natal village or home area to another, more urban setting, can cause conceptualisations of health to change [24, 25]. In Ethiopian migrant communities in the UK, it was found that at ‘home’, people would laugh daily: this was a measure of wellbeing. In the new context, they might only laugh once a month [24]. Refugee status and urbanisation led to reduced levels of wellbeing and health, as measured by happiness. Movement from a familiar context to an unfamiliar one had a considerable impact on the health and wellbeing of these Ethiopian refugees.

Conceptualisation of illness altered within new contexts. There was concern about iatrogenic disease due to different healthcare settings. Social isolation and disconnection were seen as major factors causing disease when people move away from their kin, families and support network [24], and no longer have access to the types of herbal medicines which they would use for more minor ailments at ‘home’. When communities are removed from their indigenous context, it leads to loss of indigenous herbal knowledge which can contribute to poor health outcomes [26].

In the existing literature about indigenous healing in Kenya, we find that gender is poorly represented. Typically the voices represented are those of men. This is often for good reason, but efforts should be made to create a balance in this narrative. For example, in a study of Samburu pastoralist ethnoveterinary knowledge, 29 men and only 5 women were interviewed [27]. One key aim of my research therefore was to allow space for female healers’ voices to be heard and to shed light on the wealth of knowledge held by women.

## Methodology

### Theoretical framework

The study aims to document local perspectives to understand how people themselves conceptualise illness and wellness, as well as medical choices that derived from such conceptualization. In its most basic sense, I use grounded theory to allow the data to speak rather than imposing meaning on it [28]. The study draws on ideas from critiques of integrative medicine: by approaching alternative forms of healing from a purely allopathic contextual framework, we erode its capability to exist as a radical alternative [29] although in the context presented in this paper, indigenous healing is arguably not a ‘radical’ alternative but the *only* alternative. Rather, we must reimagine alternative paths to medical care within their own frameworks, since to force these into standard biomedical contexts is to attempt to remold into a space in which it does not fit. Therefore, the study aimed to explore indigenous medicines in Kenya from individual perspectives and on its own merit, rather than comparing it to biomedical pathways to healing.

The study also considers the learnings from health equity theory: that morally, inequalities in health are unjust and unnecessary [30] often stemming from policy level issues. In this case, we will see that policy relating to safety of medicines and lack of prevention of infiltration of counterfeit medicine into local markets are critical in individual choice.

### Study site

The study focused on the concept of One Health. This is the idea that human, animal and environmental health are intimately connected and constantly intersect. I used ethnographically informed methods which include: prolonged time spent in the field (multiple visits over a period of 6 months), extensive interviews with participants and key informants, observations of medicine making and administration, and focus groups, and took extensive notes based on these interactions. This enabled better understanding of how people make choices for treating themselves and their livestock, and how medicine of different types is perceived at a community level. Fieldwork took place over 6 months, with multiple visits to the study sites during 2019 in two locations in Kenya. The rural location is in Kasigau, Taita, a semi-arid area of small-scale farmers and livestock herders in five small villages surrounding a mountain around 200 km from Mombasa. As of the 2019 census,^2^ 13,866 people live in Kasigau ward, with an average of 15 people per sq. km, and depend largely on their livestock for income and food. I have been working within this area as a researcher for around 13 years. The second is a rapidly growing densely populated area of Ongata Rongai, a suburb of Nairobi located in Kajiado county. As of the 2019 census, the population of Kajiado North sub county was 306,596, with an average of 2773 people per sq. km – one of the most densely populated areas in the country. In Rongai, many people are livestock owners, mostly of chickens and goats due to space limitations, but also engage in small businesses. Others work in central Nairobi. In Rongai I was much less familiar with the location and this was my first time conducting fieldwork there.

Given that in both study sites, many people own livestock but within a very different context, it was possible to understand how contexts might affect how people live with their animals and how they access care given different geographical, economic and social circumstances. In Rongai, participants were a mix of tribes but predominantly Kikuyu – it is an area of economic immigration from rural areas for people seeking paid work in the city. In Kasigau, the participants were either Taita or Duruma, both coastal tribes largely reliant on subsistence farming and livestock. All participants were from lower income groups.

In Rongai, there are many clinics and agrovets, access to both government and private care, and medicine is readily available. In Kasigau, access to care for livestock and humans is very poor. There is one veterinary officer and one government clinic. The nearest private clinics and hospitals are over 2 h drive away.

### Data collection

Data collection took place over a period of approximately 6 months in 2019. My methods included two focus group discussions in each study site, 40 extended unstructured interviews and key informant interviews, and observations:

#### Focus groups

Two focus groups were conducted in each study site, one with men and one with women, with an average of ten participants in each group. Participants were recruited by assistants to the chief, as is often standard practice in communities where the researcher is not well known. I specifically requested participants be from a range of economic backgrounds and different ages. Men and women were separated due to potential sensitivity of discussion topics surrounding health, and because sometimes women are not free to speak unhindered in front of their male peers. Separation of gender for focus groups allows those individuals with similar experiences to identify with each other and therefore discuss more effectively issues which they might have in common [31]. Although not traditionally ‘ethnographic’, focus groups can be effectively utilised to enhance and triangulate more traditionally ethnographic methods [32]. I facilitated these focus groups as I am fluent in Kiswahili.

#### Interviews

Extended, unstructured interviews were conducted with participants, totalling around 15 in each of the two study sites. This classically ethnographic method allowed for participants to speak freely on the subject on indigenous healing [33]. The participants were found by snowball sampling, which involved talking to one participant and being directed to another based on their social network, knowledge of other healers or individuals who had experiences of indigenous healing. Questions focused on indigenous healing types and biomedical care for both humans and livestock, ideas about access, equity and beliefs, as well as health seeking behaviour and decision making. I conducted these interviews as I did not have the need for a translator, but in Rongai my assistants located the interviewees as I did not know the area well. In Kasigau I knew most of the people who I interviewed and had gained clearance from the sub-chief, so had no need for an assistant.

Interviews were conducted with female and male healers of humans and animals, users of both indigenous and biomedical care providers, and owners of livestock, and recorded using an audio recorder and handwritten notes during interviews and conversations. I aimed to get a balance of female and male participants but in reality the split was around 60:40 in favour of men. This was partly because men practice healing on a more formal basis (in order to make a living), but also because it is accepted that women hold a certain amount of herbal knowledge as part of their role as domestic and family care giver, and this is more rarely used to generate income. Furthermore, I found that in Kasigau female healers were less forthcoming because they were afraid of witchcraft accusations which although uncommon are still a reality and a genuine fear for women. Due to the lack of representation of women healers in the literature, I aimed to give as much space to women’s voices as possible by specifically seeking out female healers wherever appropriate.

Importantly, while conducting interviews I was looking for a saturation of responses. This can be identified through a repetition of themes or responses in my interviews. This is usually achieved by conducting between 30 and 50 interviews [cite]. Once this saturation was achieved I could begin analysis. In total I conducted 30 interviews with both key informants and participants.

#### Observations

In Rongai, I was introduced by the area sub-chief to my two assistants, one male and one female. Both are well known and trusted in the community which allowed me to walk around, speak freely to people, and take photographs to illustrate daily practices of healing and medicine. I used purposive sampling and met with female and male healers of humans and livestock, was shown different types of medicines which people used, and observed herbal medicines being made, sold and administered. I visited farmers and herders at their homesteads and while out with their animals to ask about and observe how people treat and interact with their livestock [34]. I also met with small-scale commercial sellers of herbal medicines, who have kiosks from which they sell their remedies. Finally, I visited a herbal clinic which provides juice based cures for chronically ill inpatients and outpatients. There I was able to interview each admitted patient and to speak to outpatients who were returning for check-ups. Questions focussed on the history of their illness and which different forms of treatment they had accessed before attending this clinic.

In my rural study site of Kasigau there was an extended period of observation with the local veterinary officer where I went out with him on a daily basis for around 1 week and assisted him with a range of tasks. This gave me the opportunity to observe his work, but also allowed me to speak to a wide variety of farmers about their livestock, the diseases they experience, and how they decide whether to call the veterinary officer or to use indigenous or herbal remedies.

### Data analysis

I am bilingual in English and Kiswahili, and so conducted and transcribed all interviews and interactions with participants myself. Analysis of narrative data were ongoing and the questions were adjusted and informed by the existing data as the project proceeded [35], so analysis of much of the data was performed concurrently alongside data collection. Data were stored in Nvivo 11, then extracted into word documents for an open coding and thematic analysis. The themes emerged from the data through multiple readings and re-readings, and narratives were then collated and organised under these thematic headings in a separate Word document for each theme. The reading and re-reading of narrative data also allows the researcher to be intimately connected with the narratives allowing for depth of analytical engagement. The data emerging from different methodologies were triangulated to check for consistency and continuity in responses, which indicates a shared reality and therefore a degree of reliability. The use of multiple methods and an extended period of time spent in the field is essential to bringing rigour to our work as anthropologists.

### Study limitations

The nature of qualitative research allows for a certain degree of bias and therefore it is important to recognise and address these potential limitations. My positionality as a white female researcher is likely to influence levels of engagement and responses to questions, with participants keen to give the ‘right’ answers. Additionally the focus groups paid a small amount for reimbursement of any travel expenditure (equivalent to just under $2 per participant). This could have influenced willingness to take part and potentially the type of participants who attended – for example they could have been from poorer economic backgrounds and needed the money which could mean in turn that they were more likely to need access to herbal medicines (as typically they are cheaper than biomedicines). Research was conducted in majority poor communities and therefore we cannot claim that this study also reflects the views of the middle class in Kenya, although a small number of respondents were middle class. This could contribute to a picture of indigenous medicine being more widely used than in reality, and therefore has implications for the realities of health equity. However, this study is not intended to be representative of the Kenyan population and health equity nationwide: it can be seen as a snapshot of these lives at this time.

### Findings of thematic analysis of interviews and focus groups

Thematic analysis revealed the most commonly recurring themes to be trust of medicines (human and livestock, biomedical and indigenous) and health care providers, and fake/real medicines and healers, as barriers to equitable access and use. The next theme is chemicals, and unknown and untrustworthy contents of medicines. The final theme, urban vs rural, indicates that rural and urban populations both value indigenous medicine for human and livestock wellbeing.

### Ethics

I gained oral recorded informed consent for all participant interactions, explained that participants can withdraw at any time, and never recorded names of individuals anywhere to maintain anonymity. Participants were also given the opportunity to take my telephone number in case they later had any questions or wanted to redact their data. Ethical clearance was granted for the study by IREC and by the University of Liverpool Ethics Committee, as well as a NACOSTI research permit in order to undertake the study.

## Findings

### Theme one: trust

#### Of medicines and institutions

Conversations with participants indicated a strong support base for herbal and indigenous cures, and much less so for biomedicines and institutions, largely due to negative experiences. These experiences led to a lack of trust in biomedicine or biomedical providers of care:

*The problem is, people go to hospital and they are tested and then told that your illness is not visible in the blood or in the test. So then you have no option but to go and use other types of medicines, like these.* (Female indigenous healer, interview, Rongai)

Illnesses which remain undiagnosed by biomedical means have no specific treatment. This makes patients frustrated, confused and angry. Experiences of a lack of clarity and effective diagnosis at clinics and hospitals mean that more people are choosing indigenous healing as a first option, rather than a last resort, because they cannot trust that biomedical treatment is effective and without a clear diagnosis, patients prefer to use indigenous healing as they see it working on the whole body more broadly, rather than targeting a specific illness. Similarly, a lack of communication or understanding between care provider and patient led to mistrust:

*There are those who have been to hospital, and they go multiple times and they just don’t see any improvement. You see? Now, they are tired of going and seeing no change.* (Female indigenous healer, interview, Rongai)

*The problem with the hospitals is that you go and get tested and they just don’t explain anything to you. They don’t tell you what is wrong!* (Male outpatient at a herbal clinic, interview, Rongai)

Frustration due to a lack of clear communication was a core issue reported by most respondents. A lack of specific diagnosis or perceptible change led to patients seeking alternative care:

*If you go to the hospital that medicine can refuse to treat your body. And these trees [herbal medicines] can kill all the germs in your body. The ones causing that sickness. That is why people like them.* (Male indigenous healer, interview, Rongai)

*So I was tested at the hospital and they said my blood was really down [anaemic] and I was given medicine and sent home. But I didn’t see any change. I returned to hospital after two days or three days to tell them I still couldn’t eat anything. So they sent me up to the district hospital. And there they tested me and told me I had asthma. I was given medicine for asthma, because they said it was a lung problem, so they treated me and sent me home. But again after three days I hadn’t seen any improvement. So I went to another hospital also in Kitale, and they did a blood screen and told me I might have an infection. But when they got the results they said they couldn’t see anything wrong. They just kept saying my blood was down and so I was defeated because they just kept saying the same thing and there was no improvement.* (Female inpatient at a herbal clinic, interview, Rongai)

Furthermore, the lack of staff and consistency in service at government or cheaper private institutions caused frustration and scepticism:

*And then another thing, if you go to the hospital, you wait for ever and then there is no doctor around, come back tomorrow. So you try another hospital and it’s just the same. The doctor is not available.* (Male participant, focus group, Rongai)

Medicines were often feared to be counterfeit and participants reported widespread mistrust of them:

*Healer: Yes, these days, even Panadol can be fake!*

*Patient 1: yes even I saw a clip on whatsapp about Panadol. So those panadols we are taking, if you see the effect on someone’s body, it is bad. It boils the body.*

*Healer: Yes, it’s really bad for the stomach.*

*Patient 1: so we are being told to be more aware, when you go to the shop to get something like Panadol then sometimes the packaging can look different. You have to be careful. It’s not the original one. It’s just fake. They are selling us fake ones.*

*Patient 2: you can’t even know which is which with those ones. Because they look alike. But they affect you differently. These days you get the fake, and the original. Fake and original. It’s like that. The problem is even if you look closely you can’t know.* (Female healer and her two female patients, interview, Rongai)

Based on negative experiences, people were less likely to return to a biomedical setting for healthcare, and preferred to use indigenous medicine. The exception here is with emergency cases: a mother I got to know because of her enthusiasm for using herbal medicines for everyday aliments 1 day returned home to find her child had chronic diarrhoea and sickness. She rushed them to hospital. Her child received antibiotics for an amoeba, and she supplemented this with herbs to improve digestion and to reduce nausea. The decision to use herbal or biomedicine depends on the ailment, but the severity of the illness also plays an important role in this process.

In terms of veterinary medicines, negative experiences also impacted livestock owners’ willingness to trust and use agroveterinary medicine again. One farmer in Kasigau explained that he had bought what he thought he had been advised to purchase by the veterinary officer, but because he was “poor in reading” he had bought vitamins instead of antibiotics and his chickens died. He blamed this on the medicine and instead told me that he would prefer to use herbal medicine in future because it is much easier to understand how to use – “we just know [how to use it]” he said. Issues of literacy have an effect on equitability in access to the correct medicine and little provision is made for this within a biomedical or veterinary medicine context.

Other farmers in Kasigau had experienced issues with efficacy of agroveterinary medicines, for whatever reason, and so did not trust it to cure their livestock. It could be that the medicines were counterfeit or that they had used the wrong medicine or an incorrect dose, but it was generally felt that efficacy was inconsistent. In addition to this, the cost of agroveterinary medicines meant that livestock owners often preferred to use herbal medicines. The veterinary officer on a number of occasions had to persuade them to use the agroveterinary option, sometimes administering care pro bono. The expense of these medicines often means that they are out of reach for poorer farmers and livestock owners, again creating inequitable access.

#### Of doctors, healers and pharmacists

Not only did participants fear biomedicines to be counterfeit, but there was concern that even the clinicians themselves are not qualified:

*There is no equipment [in the hospital] and they [doctors] don’t have the real certificate, it is bought or it is fake somehow.* (Male participant, focus group, Rongai)

Participants reported a lack of regulation or guidelines led to concern about the authenticity and skill of indigenous healers:

*So the problem is that we often become dependent on the local healers and as we say, it’s not always possible to know which are the real ones. You can’t possibly know which are the qualified herbalists. So it would be good if the government could just do something about it so we know we are accessing the right people for medicine.* (Male participant, focus group, Rongai)

There was concern around ‘fake’ indigenous healers but the fear which accompanied this was not the same as for ‘fake’ biomedical doctors or medicines. Fake indigenous healers weren’t necessarily dangerous whereas fake biomedicines and doctors could be. Fake indigenous healers were seen as potentially problematic and a nuisance, and value was placed on those with extensive plant knowledge:

*Well one thing is maybe the healer doesn’t know the difference between the trees and they give you the wrong one. So if there is a family of plants with like 4 different ones in it, and they bring you one to use but it’s not the one which is medicine. It’s the same family, but it’s not medicine.* (Male participant, focus group, Rongai)

There is a tacit concern cited here that this is not an issue for those who can afford better quality care, and therefore that economic inequalities also create health inequalities. The issues that participants discuss here related to corruption and counterfeit medicines are clearly not an issue if one can afford to go to a top-level private hospital where genuine medicines are guaranteed.

In addition to this, participants reported considerable concern surrounding the sale of medicines over the counter at the chemists, by employees who are not medically qualified in any way. These sales seem to be largely unregulated, causing further mistrust. This problem was raised multiple times in the men’s focus group. The tacit assumption is that if you can afford to go to a top-level private clinician, you have no need to buy medicines over the counter from questionable or unverified sources. Counterfeit medicines are not an issue for the elite: indeed, in the Nairobi Aga Khan University Hospital dispensary there is a poster assuring patients that we can have confidence that their medicines are genuine.

*And because they are just a shop and not doctors they cannot advise you.* (Male participant, focus group, Rongai)

*So most of us don’t have access to effective medical care. So people are just running to the shop to get basic medicines but these medicines don’t work because often you cannot know what is wrong.* (Male participant, focus group, Rongai)

### Theme two: chemicals

Participants told me there was widespread fear of ‘chemicals’. Chemicals, they said, are found in ‘modern’ or biomedicines, both for humans and for livestock, and in every interview, conversation or focus group that I conducted during fieldwork, this was cited as a major reason for the widespread mistrust of these medicines.

*The problem is, in the clinics they have those medicines which have been mixed with all sorts of chemicals, they mix them with chemicals. Now, you know, these chemicals, they aren’t in my medicines. All those modern ones have chemicals.* (Female indigenous healer, interview, Rongai)

Ideas about chemicals in food were also raised as problematic, and people worried about ingesting ‘chemicals’ in biomedicines:

*…we know that the medicines in the hospital are made with very many chemicals. These bring many problems in your body and don’t treat you. If you go to hospital this is the type of medicine they give you. So you find many people prefer local medicines because at least they are organic. And they don’t come with so many long instructions on how to use them. The medicines from the hospital bring many problems in your body because they are full of chemicals. You end up seeing changes in your body which are bad.* (Female participant, focus group, Rongai)

As a direct response to this mistrust of biomedicines and ‘chemical’ medicines, indigenous healers allow their clients to watch them making their curatives. This seems to instil a level of trust between healer and patient which is not present with biomedical care providers. It requires an intimacy, an interaction and a sharing of space and time which is in direct opposition to the immutable space and hierarchy of knowledge experienced by patients in biomedical settings. The creation of herbal medicine in the presence of the patient is an act of sharing and knowledge equity, shunning obfuscation of process, unlike the rapid and impersonal interactions between a doctor and patient in a hospital setting:

*And [with biomedicines] you can’t know where or how they are made. But here I can sit and see how she is making them. She makes them as I watch. I see how they are made, what is going into them.* (Female patient of indigenous healer, interview, Rongai)

Equity is created in this relationship between patient and healer by taking time, talking together as neighbours and friends, and allowing for transparency in the process of producing the medicine.

Participants worried that Kenyans are being sold biomedicines which are expired and cannot be sold elsewhere:

*So you are taking that medicine but it’s not necessarily doing what you need it to. And so many are expired. You can read that in the newspaper, I had read about it, it is a real problem here.* (Male participant, focus group, Rongai)

This narrative reflects a 2019 scandal with Always pads where the quality of those sold in Kenya was found to be below the standard sold globally. This ongoing discourse of poor quality products being sold in Kenya feeds into the counterfeit biomedicine narrative:

*Yes, and also sometimes you go to buy medicine and what they are giving you is like a sample, it’s not really medicine. It’s not the original one. They substitute them and they are not the real one. Or they write you a prescription at the hospital and you go to the chemist and what they give you is not the real one, it is different.* (Male participant, focus group, Rongai)

There was a lack of confidence in government legislation to protect consumers from counterfeit medicines and their ‘chemical’ or unknown ingredients:

*Also the other thing is corruption at KEBS [Kenya bureau of standards] and so they are bringing in things with no quality because they are only looking for money so the quality of the medicines is very poor and yet it has that KEBS sticker so what can we do? So only the people at KEBS know what is really inside that medicine but the maker is going to get the stamp and they are given the KEBS stamp because they have paid for it and KEBS don’t care if it is poor quality.* (Male participant, focus group, Rongai)

### Theme three: urban and rural populations both value indigenous healing

Due to the lack of equitable access to hospitals or reliable sources of biomedicines, people are quicker to choose indigenous healing since it is readily available and cheaper. The fear or mistrust of anything ‘chemical’ was a recurrent theme in narratives in Kasigau, as it was in Rongai, perhaps even more so. However, there is not the same proliferation of biomedical care in this area so although people worried about chemicals and side effects of biomedicines, herbal medicines are widely used and more trusted because of a lack of alternatives. The one poorly served clinic in Kasigau is unable to provide quality care for patients. Good quality biomedical care can be accessed in Mombasa but this is over 4 h away by public transport; an expensive journey and only the wealthy can afford treatment in these hospitals, again contributing to unequitable access to care for this community.

*Often you take children to hospital 3 times and you see no change so you take them to local healers instead.* (Female participant, focus group, Kasigau)

*For fevers, you go to hospital but people get fed up using the same medicines day after day for weeks on end. This is why they often use local ones instead. You get better faster and the clinical medicines have side effects and chemicals and can bring allergies* (Female participant, focus group, Kasigau)

The issue of chemicals in biomedicines was also seen as an important factor in preferring to use indigenous medicines. The idea of treating like with like, as in homeopathy, was common – organic bodies should be treated with organic medicines:

*There is a lot of mistrust of clinical medicines. They bring problems which the local ones do not. They are original, no chemicals involved. Clinical medicines are not fresh, they are able to sit for so long in the dispensary or pharmacy – how is that possible without all the chemicals? Even if you look now, old people never used to get cancers, pressure, etc. They used only organic things, natural things. Now people get more sick, not like before. It is because of all these processed things*. (Female participant, focus group, Kasigau)

The problem of literacy necessitated the use of indigenous medicines as these were seen as relatively harmless:

*There are many chemicals in clinical medicines, many side effects and people get worse. Maybe it can be expired, or you use it wrong. Often the problem is you can’t read well and can’t follow the instructions. Then you get more sick. Local medicines leave no chemicals in the body. You cannot overdose on it.* (Male participant, focus group, Kasigau)

There was mistrust of biomedical treatment for livestock too, which was not mentioned in Rongai. In Rongai, use of medicines for livestock seemed to depend primarily on the type of sickness the animal had, and the ability of the owner to pay. In Kasigau, people stated a mistrust of agrovet medicines, again related to the idea of chemicals being dangerous:

*These medicines at the agrovet have a lot of chemicals in them. The local medicine is simple* (Female livestock owner, Kasigau)

The issue of fake vs real was not so pronounced in Kasigau but the issue of trust was still foremost, as indicated by these narrative extracts. Mistrust of ‘chemical’ medicines was immutable, but depending on the context, the importance of this varied. Since Kasigau is geographically remote, there is increased reliance upon herbal and indigenous healing methods and knowledge. The small and basic local clinic lacks funding and therefore lacks medicines, health care workers and equipment. There is only one agrovet in the area which is poorly stocked and rarely open, and one overworked veterinary officer covering a very large geographical area on his own personal motorbike with no diagnostic or laboratory facilities at his disposal. Unless a family is relatively wealthy, often the only choice is to use herbal curatives with mixed results for both themselves and their livestock.

For people living in the rural study site access to herbal medicine is easier than it is for those living in the urban site. However, this did not prevent urban dwellers from accessing herbs – it just meant that healers are the conduit through which these medicines must be accessed as they have a supply chain from rural areas where these plants grow. It does not necessarily tally that wealthier urban populations use indigenous medicines less due to improved access to biomedical care.

It does seem to be the case that illnesses which last longer are more likely to be treated by indigenous healers, or a combination of indigenous and biomedical. The way in which this was explained in both of the study sites was that the longer an illness lasted, the less effective the medicine seemed to be, leading to a mistrust of either the medicine itself (“it could be fake” or “it’s not the real one”) or of the diagnosis and practitioner (“they don’t know what is bringing the sickness” or “they couldn’t see anything in my blood”).

## Discussion

This study shows current trends in Kenya related to health and wellness seeking behaviours based on first-hand experiences of the local population. Such trends appear to follow global trends in these issues. In recent years, there has been a move away from biomedical medicines (especially for more minor ailments) towards organic, natural and herbal medicines and curatives [7]. The feelings and ideas of the participants in this study are reflected globally: people are concerned about what they are putting into their bodies [36]. Globally, marketing of high street wellness products shows the trend for locally-sourced, natural and organic ingredients matters to consumers [37]. Findings from my study indicate that people want to know the contents and the source of their medicines and curatives here, as several other studies [36, 37] have shown globally. The theme of ‘chemicals’ indicates the fear of putting unknown and unfamiliar medications into one’s body, and the concern that these might not even be genuine is demonstrated by the theme of ‘fakes’. These themes have implications for health equity because when patients do not trust a biomedical system of care they will be less comfortable accessing it. It may cause them to use indigenous medicines which, while the patient may be more familiar with the ingredients and the healer, there may be contraindications which they are unaware of, or the medicine may be of poor quality. To allow for more equitable interactions, all healers should demystify the process of healing, explain in clear understandable terms to the patient, and give space for questions and concerns. These two clear thematic outcomes of ‘fakes’ and ‘chemicals’ call into question the one-size-fits-all approach of allopathic medicine, indicating broad useage of indigenous medicines for many ailments in humans and livestock.

The market is responding to this and even items such as bath and body care products reflect this change as a crucial part of the wider global environmental movement. People care about what they put into and onto their bodies, especially where health is concerned. Even domestic animals in the global north can be taken to homeopathic vets when their humans no longer trust biomedical veterinary medicine, especially for prolonged illnesses, which is similarly the case for humans [8].

This trend is an important one as it effects how humans access medicines and health care for both themselves and their livestock, and therefore the global burden of disease. It has a significant bearing on equitable access to healthcare: indigenous healing is bridging the gap where there is either no clinic or vet, or where people do not trust biomedical medicines and doctors. Human and livestock health are intimately connected and that livestock are a critical part of the food chain globally and even more so in the global south [38]. In Kenya, hospitals and veterinary services are chronically under-funded [39] and people and livestock therefore are likely to suffer if this were the only means of treatment available. People are often forced to seek alternative care for themselves and their animals which is not always effective, safe or regulated.

Indigenous healers are usually able to foster more equitable relationship with their patients. They are embedded in the community, often born and raised there, and known by their peers. Medical doctors in government hospitals in Kenya can be posted from anywhere in the country, and so are not necessarily familiar with or in the community where they are practicing. This, in addition to a strong knowledge hierarchy, creates distance, misunderstanding and mistrust between medical doctors and patients. Indigenous healers on the other hand make medicines and concoctions in front of patients. This openness and transparency of knowledge production creates an egalitarian relationship between healer and patient, regardless of efficacy or regulation. It creates a relationship based on trust, because as humans we trust what we can see. We know these healers as integral people in our communities. They are our neighbours, our friends, our family, and so believe that they must have our best interests at heart. People do not know who the biomedical doctors are, and the ingredients and process of producing biomedicines are unknown. Indigenous practitioners often demystify healing and allow for trust and equity.

This study has provided insight into the everyday health seeking practices of people in Ongata Rongai, and Kasigau, for both themselves and their livestock. This addresses directly the issue of what people do when they cannot afford, access, or trust biomedical or veterinary medicines. It strongly indicates that big pharmaceutical companies have important work to do if they wish to maintain their lion’s share of the medical market. People do not trust biomedical medicines. They understandably do not want to put ‘chemicals’ and unknown mixtures of drugs into their bodies unless they really have to. People want to know where their medicine comes from, what it contains, and if it really works. They want assurances. They want to know what side effects they might cause and if they can trust the medicines to be genuine and of good quality.

Not only do the big pharma companies have work to do, but there is clearly an argument for greater government regulation of medicines, both biomedical and herbal, and effective enforcement of existing regulations. Robust regulation could increase trust and use of biomedical and herbal medicines which may lead to better health of the nation overall through improved coverage and equity of health resources. Furthermore, indigenous care providers need more support to be able to follow existing legislation for registration and regulation [8, 40, 41] in order for people to be able to distinguish between ‘fake’ and ‘real’ healers and medicines.

## Data Availability

The datasets generated and analysed during the current study are not publicly available due to content which might identify individuals within the full recorded narratives but are available from the author on reasonable request.

## Footnotes

1 A note on terminology: throughout this paper I will use the term *indigenous healing* to refer to a variety of herbal, spiritual and bone setting techniques in a broad sense, as well as similar types of treatments for livestock and domestic animals. Specific mentions of *herbal medicine* refer to those herbal concoctions produced by healers and taken by patients to treat ailments. Ethnoveterinary medicine is referred to separately using this specific term.

2 https://www.knbs.or.ke/?p=5621
